# Surgical management of bilateral concomitant posterior fracture-dislocation of the shoulder

**DOI:** 10.1016/j.tcr.2022.100691

**Published:** 2022-08-08

**Authors:** Wael Ben Fadhel, Roman Ghrairi, Sylvain Sabathe, Thierry Bégué

**Affiliations:** Department of Orthopedic, Trauma and Reconstructive Surgery, Antoine Béclère Hospital, AP-HP, Paris-Saclay University, France

**Keywords:** Bilateral, Posterior, Shoulder, Dislocation, Reverse hill-Sachs lesion

## Abstract

We present the case of a 71-year-old man who suffered a bilateral posterior fracture-dislocation of the shoulder after an idiopathic seizure.

After immediate closed reduction, CT-scan images revealed bilateral anterior reverse Hill–Sachs lesion superior to 30 % of the articular humeral head. A surgical treatment was performed for reconstruction of segmental defects of the articular humeral heads followed by filling the defect using lesser tuberosity transposition. Early rehabilitation protocol was prescribed. After 14 months, the patient returned to normal daily activities with no complaint.

Anatomical humeral head reconstruction and bone defect filling resulted in a good clinical outcome after posterior shoulder dislocation. It can be the treatment of choice for large humeral head defects, especially in younger patients with good bone stock.

## Introduction

Bilateral posterior shoulder dislocations are rare and account for 5 % to 15 % of all posterior dislocations. They result mainly from seizure attacks [1]. The anterior impaction fracture of the articular surface of the humeral head called “Reverse Hill-Sachs lesion” (RHL) is the fracture that is most frequently associated with this type of dislocation [1]. The choice of treatment depends on the stability of the shoulder and the size of the RHL [2].

## Case presentation

We report the case of a 71-year-old patient, retired engineer, right-handed, with no previous medical history, particularly no epilepsy or diabetes, who has been brought to the emergency room of our hospital for inter-scapular pain after a sudden awakening after an episode of loss of consciousness, with bilateral functional disability of both upper limbs. Neurological examination was normal.

**Fig. 1:**
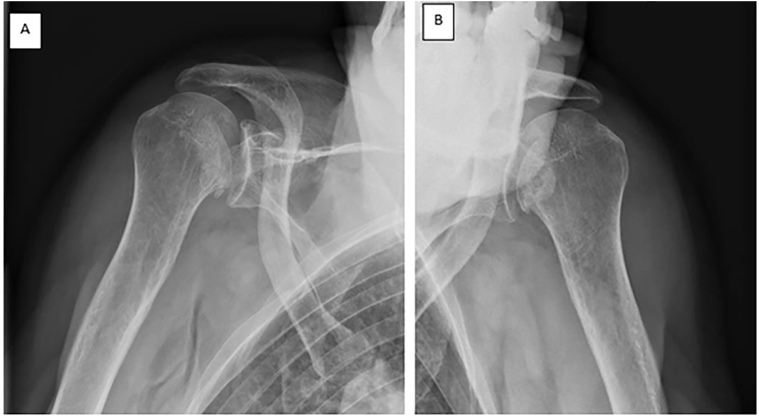
Fracture with posterior dislocation. A: Right shoulder, B: left shoulder.

The diagnosis of a first generalized seizure attack is retained (CPK 250, lateral bite of the tongue). Physical examination revealed empty glenoid fossae on both shoulders, with bilateral adduction of the upper limbs. External rotation in both arms was impossible. Emergency X-rays showed bilateral posterior shoulder dislocations with fractures of both humeral heads (Fig. 1).

The patient underwent closed reduction under short general anesthesia. After the reduction, the shoulders were unstable from 20 degrees of internal rotation. A bilateral plaster cast orthosis was applied in the safest position.

**Fig. 2:**
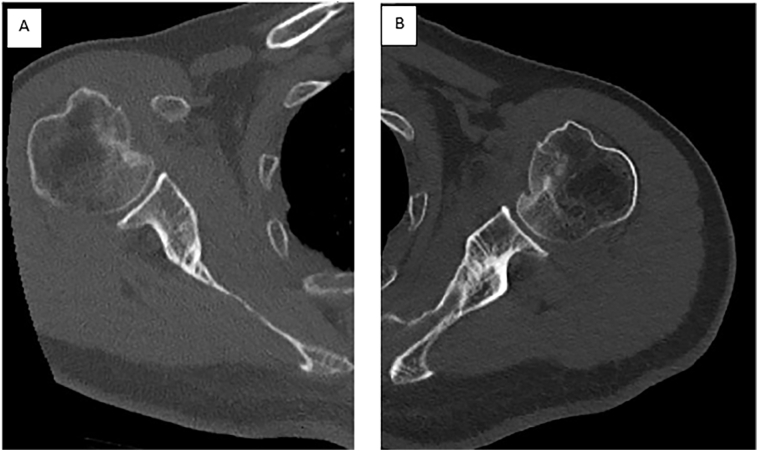
CT of both sides confirming the dislocation and measurements of Reverse Hill Sach lesion A: Right shoulder, B: left shoulder.

The CT-scan showed an anteromedial osteochondral impaction fracture of the humeral heads (Fig. 2), involving 30 % and 40 % of the total articular surface on the right and left humerus respectively, according to Cicak et al. [3]. Taking into consideration a RHL >25 % of the articular surface associated with an instability of the shoulder in internal rotation post reduction, a surgical treatment of the bilateral humeral fracture was chosen. We performed bilateral reconstruction of the humeral heads by filling the defects using lesser tuberosity transposition according to the modified McLaughlin technique described by Neer [4]. The surgery was performed on the fifth day post-injury under general anesthesia in a beach chair position. The two shoulders were operated consecutively, with the same surgical technique.

A deltopectoral approach was used, and the osteochondral defect was elevated to reduce the articular surface. Then we performed an osteotomy of the lesser tuberosity from lateral to medial starting from medial edge of bicipital groove using osteotome. Then we performed the transposition of the lesser tuberosity into the defect. Fixation was done using cannulated anteroposterior screws type Asnis® III 4.0 mm (Stryker Corporation, Kalamazoo, MI, USA) for final stabilization of the lesser tuberosity transposition. Additionally, 2.7 mm headless compression screws (Integra Lifesciences, New Jersey, USA) were used to stabilize the osteochondral fragments of the left shoulder. The shoulders were stable in maximal internal rotation at the end of surgery.

**Fig. 3:**
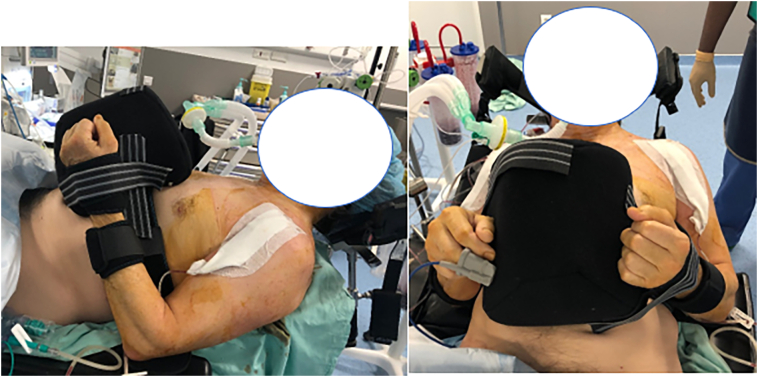
Postoperative immobilization in neutral rotation.

The patient was immobilized in neutral rotation for six weeks. An abduction pillow was used as orthosis and was placed in front of the chest to allow for bilateral external rotation of the shoulders (Fig. 3). Early rehabilitation protocol was allowed with passive range of motion exercises after three weeks. Internal rotation was prohibited during 6 weeks postoperative. Active rehabilitation was then started including active and passive range of motion, capsular stretching and muscle strengthening exercises.

The etiological assessment of the first seizure carried out. All investigations showed the absence of any structural brain abnormality. Therefore, the patient was diagnosed with idiopathic generalized epilepsy and an antiepileptic treatment was prescribed.

**Fig. 4:**
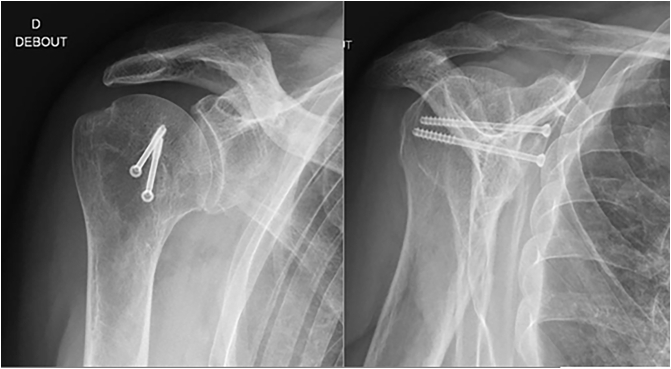
14 months follow-up X-ray Right shoulder.

**Fig. 5:**
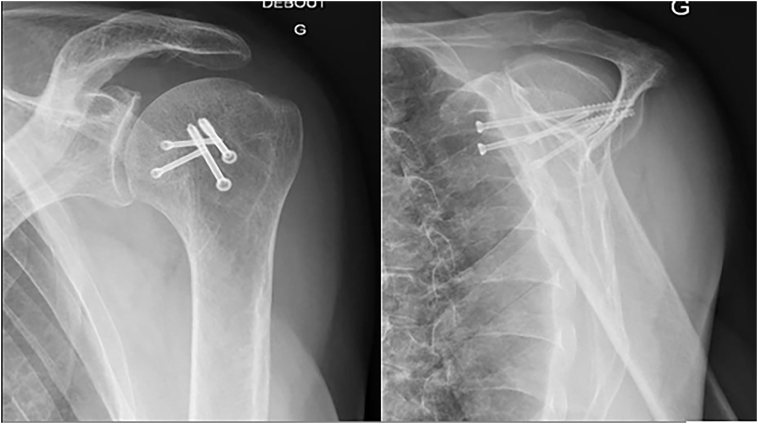
14 months follow-up X-ray left shoulder.

**Fig. 6:**
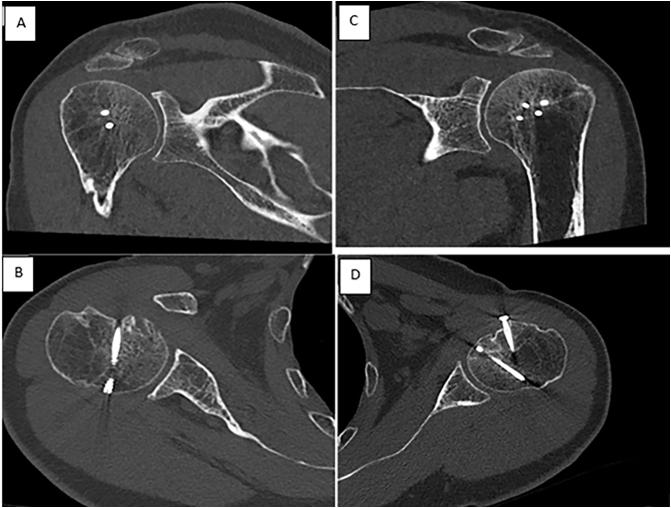
8 months follow-up CT scan. A, B: Right shoulder; C, D: left shoulder.

No postoperative complication was identified. The patient was seen at 4 months, 8 months, and 14 months postoperatively. At 14-months follow-up the patient had resumed his previous level of activity without significant discomfort (Fig. 4, Fig. 5). His Constant Score was 66 on the right side and 60 on the left side. The DASH Score was 21 on both sides and his mobility restored to a satisfactory degree (Table 1). A control CT-scan at eight months revealed a complete fusion of osteotomies and good articular shape (Fig. 6).

**Table 1:** Comparison of ROM progression and functional outcome at 6 and 14-months post-operative.

6 months					14 months			
	ROM	Constant score	DASH score	VAS	ROM	Constant score	DASH score	VAS
Right Shoulder	Flexion: 150 Abduction: 100° External rotation: 60 Internal rotation: L3	67.5	18	1	Flexion: 170 Abduction:110 External rotation: 70 Internal rotation: L1	66	21	0
Left shoulder	Flexion: 160° Abduction: 100° External rotation: 50 Internal rotation: L3	65	17	2	Flexion: 170° Abduction: 110 External rotation:60 Internal rotation: L1	60	21	0

ROM: Rang of motion; VAT: Visual analogue thermometer.

## Discussion

In case of posterior shoulder fracture-dislocation, early diagnosis helps to do an immediate CT-Scan to evaluate the humeral head impaction. When the defect of the humeral head is over 25 %, surgical treatment is needed [3], and even more indicated when the lesion is bilateral secondary to an idiopathic seizure like in our case.

If the humeral defect was <25 %, closed reduction under general anesthesia has been shown to produce good results when performed in acute cases [5]. However, closed reduction may lead to progression of the impaction defect to an anatomical neck fracture or to the displacement of fracture lines already present in the surgical neck or tuberosities, increasing the risk for avascular necrosis of the humeral head [6].

Several surgical techniques for correcting the impact of the humeral head after posterior dislocation associated with RHS Fracture have been described [3]. Nevertheless, no consensus exists due to a low level of evidence in these studies (small number of patients and lack of statistical power) and the heterogeneity and rarity of these lesions [7].

Kowalsky et Al [8] agrees that a Reverse Hill-Sach lesion ≥20–25 % and/or a subacute dislocation require an open reduction. An open reduction allows direct visualization of the glenohumeral joint, the impaction of the humeral head and an intraoperative testing of glenohumeral stability. McLaughlin [9] was the first to propose an open surgery for correction of the RHL (<45 %), by filling and transferring the tendon of the subscapularis muscle removed from the lesser tuberosity. Neer [4] modifies this technique by performing an open osteotomy of the lesser tuberosity with transfer of it into the bone defect. This modified McLaughlin procedure has gained popularity over the original technique as it is believed to offer a better bone to bone union and has produced good results in several studies [9].

**Fig. 7:**
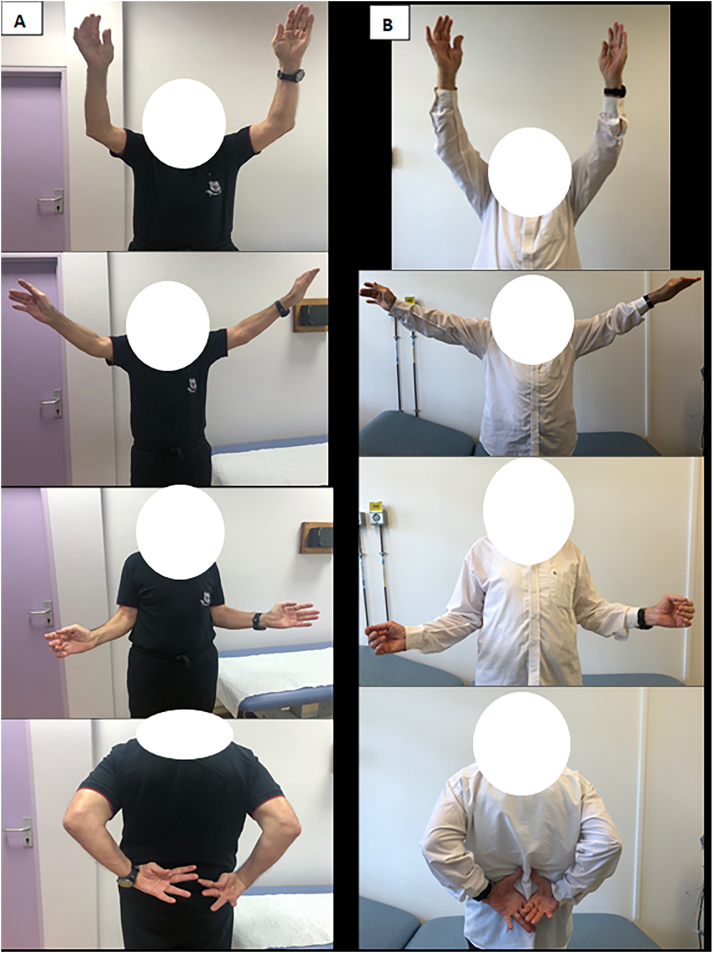
Functional outcome; A: 6 months postoperatively; B: 14 months postoperatively.

Gerber et al. [10], in his study including 19 shoulders with a follow-up of 128 months (range, 60–294 months), treated his patients by anatomical humeral head reconstruction and autograft bone-defect filling. They found similar results between their technique and lesser tuberosity transfer. In cases of defects over 35%of the articular surface, the results were better. At final follow-up, mean relative CS was 84 % and 15 out of 19 patients indicated that they had no pain (Fig. 7).

We have chosen the anatomical humeral head reconstruction associated to Mclaughlin technique modified by Neer, on both sides, despite the presence of a 40 % RHL in the left shoulder. Additional clinical findings support our choice: a) absence of glenohumeral osteoarthritis; b) absence of displaced three or four-part fractures; c) presence of a good bone stock. Our results are comparable with the literature in terms of rapid range of motion recovery as well as the CS, and autonomy recovery [11]. The restoration of the sphericity of the humeral head is a key factor for a favorable clinical outcome [11].

## Conclusion

We present a rare case of bilateral posterior shoulder dislocation after idiopathic seizure. Physical examination and radiographic evaluations are important for diagnosis of shoulder stability. If the diagnosis is made early and the humeral head impaction defect is higher than 25 %, surgical treatment is indicated. This procedure associated to bone defect filling could emerge as the treatment of choice for larger defects especially in young patients with good bone stock.

## References

[1] Rouleau D.M., Hebert-Davies J. (2012 Apr). Incidence of associated injury in posterior shoulder dislocation: systematic review of the literature. J. Orthop. Trauma.

[2] Cooke S.J., Hackney R.G. (2005). Bilateral posterior four-part fracture-dislocations of the shoulders following electric shock: a case report and literature review. Injury Extra.

[3] Cicak N. (2004 Apr). Posterior dislocation of the shoulder. J. Bone Joint Surg. (Br.).

[4] Hughes M., Neer C.S. (1975 Aug). Glenohumeral joint replacement and postoperative rehabilitation. Phys. Ther..

[5] Duralde X.A., Fogle E.F. (2006 Nov-Dec). The success of closed reduction in acute locked posterior fracture-dislocations of the shoulder. J. Shoulder Elb. Surg..

[6] Kokkalis Z.T., Iliopoulos I.D., Antoniou G., Antoniadou T., Mavrogenis A.F., Panagiotopoulos E. (2017 Apr). Posterior shoulder fracture-dislocation: an update with treatment algorithm. Eur. J. Orthop. Surg. Traumatol..

[7] Robinson C.M., Aderinto J. (2005 Mar). Posterior shoulder dislocations and fracture-dislocations. J. Bone Joint Surg. Am..

[8] Kowalsky M.S., Levine W.N. (2008 Oct). Traumatic posterior glenohumeral dislocation: classification, pathoanatomy, diagnosis, and treatment. Orthop. Clin. N. Am..

[9] McLaughlin H.L. (1952). Posterior dislocation of the shoulder. J. Bone Joint Surg. Am..

[10] Gerber C., Catanzaro S., Jundt-Ecker M., Farshad M. (2014). Long-term outcome of segmental reconstruction of the humeral head for the treatment of locked posterior dislocation of the shoulder. J. Shoulder Elb. Surg..

[11] Gurzì M.D., De Meo D., Pugliese M., Di Giorgio L., Persiani P., Villani C. (2017 Dec). Bilateral posterior fracture-dislocation of the shoulder after epileptic seizure. Trauma Case Rep..

